# Network Pharmacology and Molecular Docking to Explore the Mechanism of Compound Qilian Tablets in Treating Diabetic Retinopathy

**DOI:** 10.2174/0115734099298932240308104437

**Published:** 2024-03-15

**Authors:** Jiangwei Jia, Bo Liu, Xin Wang, Fenglan Ji, Fuchun Wen, Lianlian Song, Huibo Xu, Tao Ding

**Affiliations:** 1 Pharmacodynamic and Toxicological Evaluation Center, Jilin Academy of Chinese Medicine Sciences, Changchun, Jilin, China;; 2 School of Pharmaceutical Sciences, Changchun University of Chinese Medicine, Changchun, Jilin, China

**Keywords:** Diabetic retinopathy, compound qilian tablets, network pharmacology, molecular docking, mechanism, experimental verification

## Abstract

**Background:**

Diabetic Retinopathy (DR) is one of the common chronic complications of diabetes mellitus, which has developed into the leading cause of irreversible visual impairment in adults worldwide. The Compound Qilian Tablets (CQLT) were developed in China for the treatment and prevention of DR, but their mechanism of action still needs to be clarified.

**Objectives:**

In the present study, network pharmacology, molecular docking, and *in vivo* validation experiments were used to investigate the active components and molecular mechanisms of CQLT against DR.

**Methods:**

The active components and targets of CQLT were collected through the TCSMP database, and the targets of DR were obtained from GeneCards, OMIM, and Drugbank databases. We established a protein-protein interaction network using the STRING database. Gene Ontology (GO) and Kyoto Encyclopedia of Genes and Genomes (KEGG) pathway enrichment analyses were conducted using the Metascape database. Molecular docking using AutoDock Vina was performed to investigate the interactions between components of CQLT and core targets. Moreover, we selected ZDF rats to establish a DR model for the experimental studies.

**Results:**

39 active components and 448 targets in CQLT were screened, among which 90 targets were shared with DR. KEGG pathway enrichment analysis identified 181 pathways. The molecular docking results demonstrated that the main active components had strong binding ability to the core targets. The results from animal experiments indicate that the mechanism of CQLT against DR is associated with inhibiting the retinal mTOR/HIF-1α/VEGF signaling pathway, alleviating the inflammatory response, suppressing retinal neovascularization, and protecting the function and morphology of the retina.

**Conclusion:**

The present study preliminarily explored the mechanism of CQLT in treating DR and demonstrated that CQLT exerts anti-DR effects through multiple components, multiple targets, and multiple pathways. These findings suggest that CQLT shows promise as a potential therapeutic agent for DR and could contribute to developing novel treatments.

## 1 INTRODUCTION

The prevalence of Diabetes Mellitus (DM) has considerably increased in recent years due to various causes, such as dietary changes, population aging, and environmental changes. According to a recent report by the International Diabetes Federation, the global prevalence of DM in adults aged 20-79 has reached 9.3% (approximately 463 million people); by 2045. This proportion is projected to increase to 10.9% (about 700 million people) [1]. The number of DM patients in China will rank first globally. Compared with urban areas, patients with DM in rural areas of China have a significantly higher risk of developing diabetic retinopathy (DR) [2, 3].

Although DM can damage areas of the eye other than the retina, DR continues to be the primary way that DM affects visual impairment [4]. DR is the most common and severe ocular complication and the leading cause of impaired vision and blindness. One third of the 246 million DM patients in the population have symptoms of DR [5]. The quality of life for those with DM is seriously compromised by the existence of DR, placing a heavy financial burden on society. The correlation between DR and future occurrences of cerebrovascular accidents, myocardial infarctions, and congestive heart failure is noteworthy [6]. Notably, DM patients experience pathophysiological alterations within the retina that impact their visual function even before the emergence of microvascular lesions in the fundus. Therefore, early diagnosis is optimal for averting or delaying visual impairment and reducing associated expenses. Regrettably, therapeutic measures targeting these early-stage impairments are currently lacking [7, 8]. It can be challenging for patients to recognize symptoms in the early stages of DR since they are either nonexistent or mild [9]. When symptoms manifest in the advanced stages of DR, irreversible changes typically take hold. Therefore, it holds considerable clinical significance to initiate the prevention and treatment of DR during the early stages of DM progression [10].

Over millennia, Traditional Chinese Medicine (TCM) has provided a reservoir of knowledge and practices in addressing DM complications [11]. In recent years, many studies have confirmed that some TCMs and their active ingredients are essential in treating DM and its complications [12]. In treating DR, TCM adopts an integrative approach to prevention and treatment by profoundly exploring the pathogenesis and comprehensively regulating patients' bodily functions. Additionally, TCM treatment for DR has the advantages of being simple and convenient. Most TCM preparations are oral formulations, which leads to higher patient compliance. The pathogenesis of DR is extraordinarily complex, which means that it is difficult to comprehensively deal with the disease using any single, concise, or direct treatment mechanism [13]. Moreover, TCM is often complex herbal extracts containing multiple components, which can act on multiple targets and through multiple pathways. Therefore, the treatment characteristics of TCM match well with the complicated pathogenic mechanisms of DR [14]. Compound Qilian Tablets (CQLT) are composed of Panax notoginseng (Sanqi), Coptis chinensis (Huanglian), Astragalus membranaceus (Huangqi), and Rehmannia glutinosa (Dihuang). Panax notoginseng has been found to prevent and treat DR through its antioxidant and anti-inflammatory effects [15, 16]. Coptis chinensis can relieve and treat DM and its complications by reducing oxidative stress and regulating mitochondrial function [17]. Astragalus membranaceus can inhibit Vascular Endothelial Growth Factor (VEGF) production and positively affect DR [18, 19]. Rehmannia glutinosa and its compatible applications have a long history. Many clinical trial data have proved that it has excellent anti-inflammatory and anti-tumor pharmacological activities and has rich practical experience in preventing and treating DM and its complications [20, 21].

CQLT contains multiple components that may collaboratively exert therapeutic effects on DR by acting on various targets and pathways. Due to the diversity of the components of this formula, the key active components and mechanism of action for the treatment of DR have not been elucidated. Network pharmacology is an analytical approach based on systems biology theory, which can be used to evaluate the relationships between drugs, active components, targets, pathways, and diseases [22, 23]. In this study, we explored the mechanism of action and key active components of CQLT in the treatment of DR through network pharmacology, molecular docking, and animal experimental validation; we hope to provide a theoretical basis and experimental foundation for the clinical application of CQLT in the treatment of DR and its therapeutic mechanism development.

## 2 MATERIALS AND METHODS

### 2.1 Collection of CQLT Components

Traditional Chinese Medicine Systems Pharmacology Database and Analysis Platform (TCMSP, https://tcmsp-e.com/index.php) collects information on the components, pharmacokinetic data, and targets of various TCMs. Renowned for its authoritative and extensive dataset, TCMSP is one of the most frequently employed databases for selecting and identifying bioactive components and associated targets within TCM [24]. With the screening conditions set as oral bioavailability (OB) ≥ 30% [25] and drug-likeness (DL) ≥ 0.18 [26], the active components of Panax notoginseng, Coptis chinensis, Astragalus membranaceus, and Rehmannia glutinosa in CQLT were retrieved from the TCMSP database.

### 2.2 Target Prediction and Establishment of the Herb-component-target Network

The TCSMP database was used to screen the active component targets. The SMILES format of the active components was obtained from the PubChem database (https://pubchem.ncbi.nlm.nih.gov/). SMILES were input into the SwissTargetPrediction (http://www.swisstargetprediction.ch) to supplement targets of the active components [27]. The screened targets were imported into the UniProt database (https://www.uniprot.org/) to standardize the gene symbols of the targets. Cytoscape can create powerful visual mappings, visualize complex network relationships, and highlight the importance of each unit in the network and other practical functions [28]. Subsequently, a herb-component-target network was constructed using Cytoscape 3.10.0.

### 2.3 Collection of Targets for DR

The keyword “diabetic retinopathy” was used to search for relevant targets from GeneCards (https://www.genecards.org/) [29], OMIM (https://omim.org/) [30], and Drugbank (https://go.drugbank.com/) [31] databases. Targets with a *Relevance score* > 10 were selected in the Genecards database, and these targets were merged with those collected from the OMMI and Drugbank databases. After removing duplicates, these targets were used as disease targets for this study.

### 2.4 Construction of the Protein-protein Interaction Network

Conducting an intersection analysis between the target proteins of CQLT and DR, we subsequently constructed a Venn diagram to visualize the common targets. The intersection between them is considered as a potential target for CQLT in the treatment of DR. These common targets were input into the STRING database (https://string-db.org/) [32] with the species limited to *Homo sapiens* and the minimum required interaction score set to medium confidence (0.4) to obtain a Protein-Protein Interaction (PPI) network of the key targets. Additionally, further analysis of this network was carried out using Cytoscape 3.10.0. The NetworkAnalyzer tool was used for topology analysis. Degree, Betweenness Centrality (BC), and Closeness Centrality (CC) were used as reference criteria to screen the core targets of CQLT against DR.

### 2.5 Enrichment Analysis and Component-target-pathway Network Construction

Gene Ontology (GO) and Kyoto Encyclopedia of Genes and Genomes (KEGG) pathway enrichment analyses for the key targets were performed using the Metascape database (https://metascape.org/) [33]. The organism was set as *Homo sapiens,* and the screening threshold was *P* < 0.01. GO functional enrichment analysis included Biological Process (BP), Cellular Component (CC), and Molecular Function (MF). We selected the top 10 GO terms and the top 20 KEGG pathways with the smallest *P* value and imported them into the bioinformatics map website (http://www.bioinformatics.com.cn/) for visualization. A comprehensive study of the biological characteristics and regulatory pathways of CQLT in treating DR. To further analyze the relationships between the components, targets, diseases, and KEGG pathways, a component-target-pathway network was constructed using Cytoscape 3.10.0. Use the NetworkAnalyzer tool to analyze the topological properties of the network.

### 2.6 Molecular Docking

The 3D structure of the active components with the top 10 degree values in CQLT was downloaded in the PubChem database [34] as ligands for docking. The core targets of this study were used as docked receptors, and the protein structure files were downloaded from the PDB database (https://www.rcsb.org/). The SDF format file of the ligand was converted to PDB format using Open Babel GUI software [35]. The protein was preprocessed by removing water and the original ligand using PyMOL software, then hydrogenated in AutoDockTools software, and finally saved as a PDBQT format file. Firstly, the ligand is hydrogenated by AutoDockTools software, and the charge is added automatically. The twisted bond in the molecule is detected and defined and finally exported to a PDBQT format file. AutoDock Vina software is used to perform molecular docking in a three-dimensional grid box wrapped in an active pocket, and the binding activity between the main active ingredient and the core target is verified by binding energy [36]. Finally, the docking results were visualized using PyMOL and Maestro 13.5 software to gain a comprehensive insight into the docking outcomes.

### 2.7 Materials

CQLT (Lot: 210305T) was produced by Jilin Yatai Yongantang Pharmaceutical Co., Ltd. (Changchun, China). IL-6 (Lot: A27033032) and TNF-α (Lot: A25033030) ELISA kits were purchased from Cusabio (Wuhan, China). The primary antibodies mTOR antibody (sc-517464), HIF-1α antibody (sc-13515), and VEGF antibody (sc-7269) were purchased from Santa Cruz Biotechnology, Inc (Dallas, TX, United States); IL-6 antibody (DF6087) and TNF-α antibody (AF7014) were obtained from Affinity Biosciences (Jiangsu, China). The secondary antibodies, Mouse-IgG1 (sc-525408) and Mouse-IgGk (sc-516102), were purchased from Santa Cruz Biotechnology, Inc; IgG (SA00001-2) was obtained from Proteintech (Wuhan, China).

### 2.8 Animal Experiments

Specific Pthogen-Free (SPF) grade male Zucker diabetic fatty (ZDF, fa/fa) rats aged 8-9 weeks and weighing 280-320 g, as well as SPF grade male ZDF (fa/+) rats aged 8-9 weeks and weighing 190-230 g, were purchased from Vital River Laboratory Animal Technology Co., Ltd. (Beijing, China). The animals were housed in the SPF Animal Laboratory of Jilin Academy of Chinese Medicine Sciences (Changchun, China) under controlled conditions of 20-25°C temperature, 50-60% relative humidity, and a 12 h light/dark cycle with free access to food and water. The ZDF (fa/fa) rat is a pertinent animal model for spontaneous DM and obesity, effectively mirroring the pathogenic progression of human DR [37, 38]. The ZDF (fa/+) rats were normal controls from the same strain as the ZDF (fa/fa) rats. ZDF (fa/fa) rats were fed with K5008 feed, and ZDF (fa/+) rats (control group, n=12) were fed with ordinary maintenance feed for adaptive feeding. After adaptive feeding for 3 weeks, ZDF (fa/fa) rats with fasting blood glucose > 16.7 mmol/L measured by two consecutive blood samples from the tail vein could be used as DM models. The 30 ZDF (fa/fa) rats that were successfully modeled were divided into the model group (n=17) and the CQLT group (n=13) according to the principle of blood glucose balance. The CQLT group received oral administration of CQLT at a dosage of 0.337g/kg once daily for 8 weeks. The control and model groups were administered an equivalent volume of purified water daily. In the 6th week of administration, 1 rat from the control group and 2 rats from the model group were randomly selected for pathological retina examination. Compared with the control group, the retinas of the model rats showed exudation, microvascular proliferation, and cellular vacuolar degeneration, indicating successful DR modeling. During the experiment, two rats in the model group died naturally; ultimately, 11 rats in the control group, 13 rats in the model group, and 13 rats in the CQLT group were used in subsequent studies. After 8 weeks of treatment, the rats were anesthetized, and blood samples were collected from the abdominal aorta; serum was separated and stored at -80°C for later use. The eyeballs were rapidly enucleated and fixed in a formalin solution. All animal experiments were reviewed and approved by the Animal Ethics Committee of the Jilin Academy of Chinese Medicine Sciences, with the ethical approval number JLSZKYDWLL 2018-003.

### 2.9 ELISA Assay

After allowing the blood samples to clot at room temperature for 1 hour, serum was separated by centrifugation at 4°C and 3000 rpm for 10 min and then stored at -80°C for later use. The serum levels of TNF-α and IL-6 were quantified using commercially available assay kits. The implementation of the experimental procedures strictly adhered to the stipulated protocols provided by the respective assay kit manufacturers.

### 2.10 Retinal Histopathological Examination and Thickness Measurement

The rat eyeballs were washed, dehydrated, paraffin-embedded, sectioned, and stained with Hematoxylin and Eosin (HE). Histopathological changes were observed under a 400x microscope (Olympus Corporation, Japan). Using the NIS-Elements BR image analysis system (Nikon, Japan), we measured the retinal thickness values at the central region on both sides of the optic nerve within the cross-sectional area. Two sites were measured for each animal and averaged for statistical comparison.

### 2.11 Immunohistochemical Analysis of the Retina

After the retinal sections were prepared, deparaffinized, and rehydrated, they were washed 3 times with phosphate-buffered saline (PBS) (pH 7.4), 3 minutes each time. Each section was spiked with 50 μl of hydrogen peroxide blocking solution, incubated for 10 minutes at room temperature to block endogenous catalase activity, and rinsed three times with PBS for 3 minutes. Antigen retrieval was performed on the tissue sections using citrate buffer. 100 μl of 5% Bovine Serum Albumin (BSA) solution was added to each section and incubated for 30 minutes at room temperature. Sections were sequentially subjected to primary antibody incubation, washing, secondary antibody incubation, and washing. Subsequently, 100 μl of freshly prepared DAB solution was added, and the sections were dried by gradient alcohol dehydration and sealed with neutral resin. In addition, the control group was replaced with PBS for primary antibody, following the same steps as before. Finally, the stained positive substances were observed under the microscope, and the average optical density values were measured.

### 2.12 Statistical Analysis

Data were analyzed using GraphPad Prism 9.5 statistical software, and comparisons between two groups were made by t-test, with *P* < 0.05 indicating that the differences were significant and statistically significant. The experimental data were expressed as mean ± SD.

## 3 RESULTS

### 3.1 Active Components and Targets of CQLT

Active components in CQLT with OB ≥ 30% and DL ≥ 0.18 were screened using the TCSMP database, wherein 8, 18, 14, and 2 active components were derived from Panax notoginseng, Coptis chinensis, Astragalus membranaceus, and Rehmannia glutinosa, respectively. After merging the active components and removing the duplicates, a total of 39 active components were finally obtained. 206 targets of CQLT components were collected through the TCSMP database; in conjunction with the 289 targets screened from the SwissTargetPrediction database, 448 unique targets were obtained after removing duplicates.

### 3.2 Herb-component-target Network Construction

A herb-component-target interaction network was constructed using Cytoscape software to demonstrate the relationship between active components and their targets in CQLT. This network contains 491 nodes and 1293 edges, as detailed in Fig. (**1**). This result indicated that CQLT exerted broad potential pharmacological effects through complex interactions among its multiple components and targets. Additionally, Table **1** lists the top 10 most important active components in CQLT. Details of the herb-component-target network are provided in Supplementary Material **1**.

### 3.3 Screening of DR Targets

There were 623 DR-related targets with a Relevance score > 10 in the GeneCards database, and 233 and 3 DR-related targets were retrieved in the OMIM and Drugbank databases, respectively. After combining these data and removing duplicates, a total of 803 DR-related targets were obtained.

### 3.4 PPI Network Construction

**Fig. (2):**
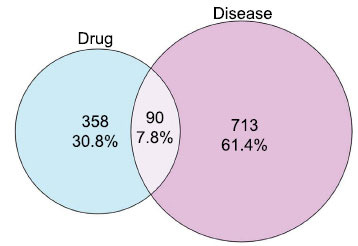
Venn diagram of CQLT targets and DR targets.

The intersection of CQLT targets and DR targets resulted in 90 shared targets. The relationship between these intersecting targets is illustrated using a Venn diagram (Fig. **2**). These 90 targets were analyzed using the STRING database to obtain the PPI network. The PPI network was subsequently imported into Cytoscape 3.10.0, generating a visualized network comprising 89 nodes and 3146 edges, as demonstrated in Fig. (**3**). The top 20 targets of degree value include IL6, TNF, AKT1, VEGFA, PPARG, TP53, IL1B, CASP3, MMP9, PTGS2, CCL2, STAT3, CXCL8, NOS3, IL10, HIF1A, CTNNB1, PPARA, ESR1, and MTOR. Detailed information on the PPI network is provided in Supplementary Material **2**.

### 3.5 GO Enrichment Analysis and KEGG Pathway Enrichment Analysis

**Fig. (5):**
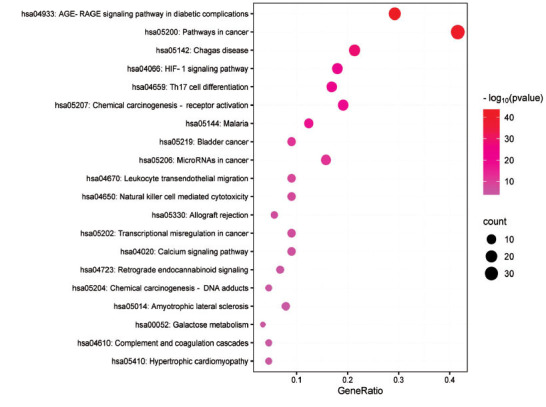
The top 20 pathways of KEGG enrichment analysis.

GO enrichment and KEGG pathway analysis of key targets were performed using the Metascape database. GO enrichment analysis yielded 1597 BPs, 48 CCs, and 114 MFs, of which BPs were mainly associated with responses to hormones, positive regulation of cell migration, response to molecules of bacterial origin, and inflammatory response. Following the minimum *P* value principle, the top 10 BP, CC, and MF entries were visualized as bubble charts, as shown in Fig. (**4**). Detailed results of GO analysis are provided in Supplementary Materials (**3**-**5**). KEGG pathway enrichment obtained 181 pathways. To illustrate the potential mechanisms of CQLT in treating DR, the top 20 pathways were visualized in ascending order of *P* values (Fig. **5**). The enrichment results indicated that the potential signaling pathways for CQLT treatment of DR were mainly related to the AGE-RAGE signaling pathway in diabetic complications, the HIF-1 signaling pathway, and the calcium signaling pathway. Detailed results of KEGG analysis are provided in Supplementary Material **6**.

### 3.6 Component-target-pathway Network Construction

**Fig. (6):**
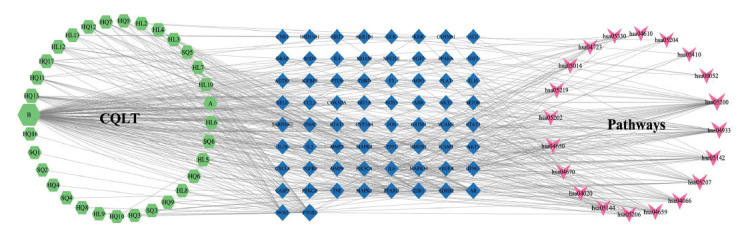
Component-target-pathway network. The blue diamond nodes represent targets, the green hexagonal nodes represent relevant components of CQLT, and the red V-shaped nodes represent the KEGG signaling pathways.

A component-target-pathway network was constructed based on the top 20 KEGG pathways with the lowest *P*-values. This network comprised of 120 nodes and 516 edges, including 66 key targets and 32 active components (Fig. **6**). Quercetin, kaempferol, and formononetin are active components with the highest degree of connectivity within this network. Moreover, IL-6, TNF, VEGFA, HIF-1A, and mTOR are core targets in this network that link active components to DR-related pathways. These results demonstrated that CQLT affects DR through multiple components, multiple targets, and multiple pathways.

### 3.7 Molecular Docking

**Fig. (8):**
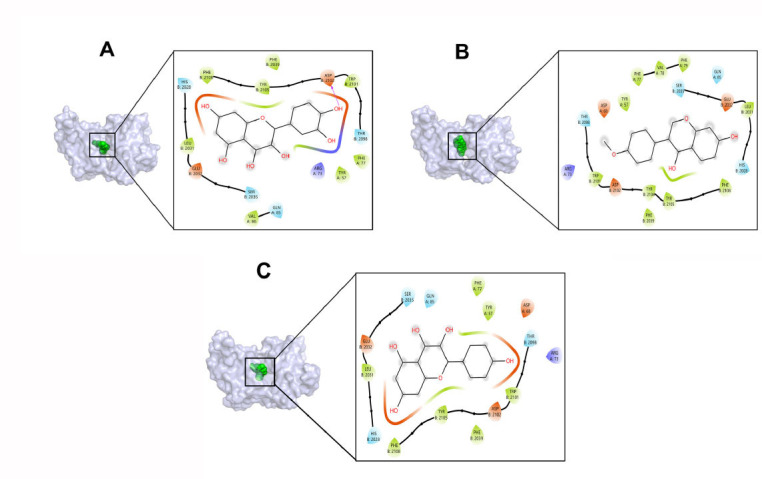
Molecular docking patterns of active components with core targets. **(A)** quercetin-MTOR; **(B)** formononetin-MTOR; **(C)** kaempferol-MTOR.

The top 10 components ranked by degree values in the herb-component-target network served as ligands, with IL-6 (PDB ID:1ALU), TNF (PDB ID:5UUI), VEGFA (PDB ID:1MKK), HIF-1A (PDB ID:1LM8), and MTOR (PDB ID:4DRI) as receptors, for molecular docking in AutoDock Vina software. The smaller the binding energy value in molecular docking, the more stable the intermolecular binding is, and the binding energy < -5.0 kcal·mol^-1^ indicates a better intermolecular binding activity [39]. According to the docking results, it was found that the docking binding energies were all ≤ -6.0 kcal·mol^-1^, which indicated that the active components of CQLT had good binding ability to the core targets [40], as demonstrated in Fig. (**7**). The molecular docking results of the key components and core targets were visualized using PyMOL and Maestro 13.5 software (Fig. **8**).

### 3.8 CQLT Reduced Serum IL-6 and TNF-α Levels in DR Rats

The results of the ELISA assay showed that compared with the control group, the levels of IL-6 and TNF-α in the model group were significantly increased; compared with the model group, the levels of IL-6 and TNF-α in the CQLT group were significantly reduced, as illustrated in Fig. (**9**).

### 3.9 Histopathological Examination and Thickness Measurement of Retinal Tissue

**Fig. (10):**
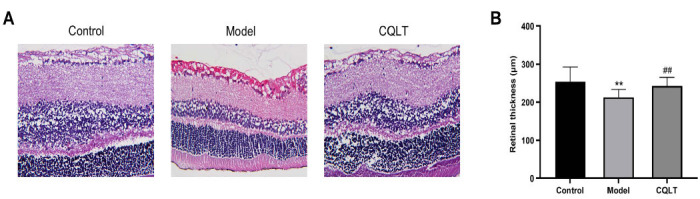
The therapeutic effect of CQLT on DR. **(A)** HE staining of rat retinal tissue in each experimental group. The magnification was 400×. **(B)** Effect of CQLT on retinal thickness in DR rats. **P < 0.01 *vs*. the control group; #*P* < 0.05 *vs*. the model group.

To investigate the effect of CQLT on retinal pathology in DR rats, we performed HE staining on retinal sections of rats, as demonstrated in Fig. (**10A**). The retinas of control rats were structurally normal and aligned, and no pathological changes were observed. Compared to the control group, the model group showed increased retinal exudation, with prominent leakage in the inner nuclear layer. Additionally, abnormal neovascularization and reduced cell numbers in the inner and outer granular layers were observed, along with vacuolar degeneration of cells in the model group. Compared to the model group, the CQLT group showed inhibited retinal neovascularization and exudation, less vacuolar degeneration of cells, and significantly increased cell numbers in the inner and outer granular layers. These results indicate that CQLT suppressed retinal exudation, microvascular proliferation, and cellular vacuolar degeneration in DR rats, normalizing retinal structure and function. Concurrently, retinal thickness was measured, and the results demonstrated that CQLT significantly increased retinal thickness in DR rats (Fig. **10B**).

### 3.10 CQLT Inhibited the Expression of mTOR, HIF-1α, VEGF, IL-6, and TNF-α in the Retina of DR Rats

The effects of CQLT on the expression of VEGF, mTOR, HIF-1α, IL-6, and TNF-α in the retina of DR rats were analyzed by immunohistochemistry, and the results are demonstrated in Fig. (**11A**). The model group rats had significantly elevated mTOR, HIF-1α, VEGF, IL-6, and TNF-α in the retina compared to the control group. This suggests that DR leads to activation of the mTOR/HIF-1α/VEGF signaling pathway and elevation of inflammatory cytokine levels in the body. After continuous administration for eight weeks, the CQLT group rats showed significantly decreased retinal expression of VEGF, mTOR, HIF-1α, IL-6, and TNF-α compared with the model group, as demonstrated in Fig. (**11B**).

## 4 DISCUSSION

Driven by factors such as diet, hypertension, hyperlipidemia, overweight, and genetic predisposition, the DM patient population is expanding rapidly [41, 42]. This also means that more people with DM will be plagued with DR. The newly released Chinese guidelines for Type 2 DM indicate that DR has become the primary cause of blindness among Chinese adults. DR primarily damages the retinal microvascular system, disrupting the blood-retinal barrier and retinal leakage, resulting in impaired vision; not intervening may lead to vitreous hemorrhage, retinal detachment, and ultimately blindness [43-45]. The pathogenesis of DR mainly involves inflammation, retinal nerve damage, oxidative stress, autophagy, retinal thickness, and vascular dysfunction [46-48]. The treatment of DR should focus on pathogenesis and provide appropriate interventions and effective treatments to control the onset and progression of DR before irreversible damage to vision occurs. Retinal laser photocoagulation, vitrectomy, and anti-VEGF therapy are currently the mainstay of treatment for DR [49, 50]. However, these treatments are limited and aggressive. Therefore, the development of economical and safe TCM and the study of their mechanism of action are of great significance in preventing and treating DR.

In recent years, China has been actively promoting the use of TCM to prevent and treat DR. As a traditional medical system, TCM has a long history and rich experience in the treatment of DR. TCM emphasizes overall balance and individual differences, focusing on the root cause of the disease and overall regulation, and therefore has unique advantages in the treatment of DR. Herbs with the functions of clearing heat and detoxifying, promoting blood circulation and removing blood stasis, nourishing Yin and tonifying kidney are often used in TCM formulations to regulate the balance of Yin and Yang in the body and promote the recovery of microcirculation; thereby improving the blood supply to the retina, promoting vision recovery, and avoiding retinal detachment. Based on systematic TCM theories and clinical medication experience, our team selected Panax notoginseng, Coptis chinensis, Astragalus membranaceus, and Rehmannia glutinosa to form CQLT. This formulation aims to introduce a novel approach to treating DR.

To investigate the crucial components and action mechanisms of CQLT for treating DR, we employed network pharmacology, molecular docking, and experimental validation to conduct the respective studies. Screening identified 39 active components and 448 targets in CQLT. Subsequently, a herb-component-target network containing 491 nodes and 1,293 edges was constructed, demonstrating that the pharmacological effects of CQLT arise through interactions between its multiple active components and their targets. Moreover, we constructed a PPI network by subjecting the shared targets of CQLT and DR to PPI analysis. The key targets were analyzed for GO enrichment and KEGG pathway enrichment, and a component-target-pathway network was also constructed. Quercetin, kaempferol, and formononetin were the top 3 components in this network. Quercetin is a natural polyhydroxyflavonoid with a wide range of pharmacological effects, including anti-inflammatory, antioxidant, anti-apoptotic, and immunomodulatory effects [51, 52]. Quercetin significantly increased the thickness of the retinal cell layer and the number of ganglion cells, and reduced the overexpression of pro-inflammatory factors Interleukin-6 (IL-6) and Tumor Necrosis Factor-alpha (TNF-α) in the retina of DR rats, which also showed some inhibitory effects on the overexpression of VEGF in the retina of DR rats [53, 54]. Quercetin also showed advantages in preventing and treating neurodegeneration in DR by improving neurotrophic factor levels and inhibiting neuronal apoptosis in rats with DR [55]. Kaempferol is a flavonoid with anti-cancer, anti-infection, and anti-inflammatory effects on DM management [56, 57]. Kaempferol has been demonstrated to inhibit the activation of the Src-Akt1-Erk1/2 signaling pathway in human retinal endothelial cells by targeting VEGF and PGF, which leads to the inhibition of angiogenesis [58]. Kaempferol inhibited the elevated VEGF mRNA expression level triggered by H2O2 in ARPE-19 cells. Meanwhile, kaempferol affected the oxidative and antioxidant homeostatic system of ARPE-19 cells after H2O2 induction by regulating the activities of Reactive Oxygen Species (ROS) and Superoxide Dismutase (SOD) [59]. These experimental results verified the potential pharmacological effects of kaempferol in the treatment of DR. Formononetin is an isoflavone compound with anti-inflammatory and antioxidant properties. It inhibits retinal neovascularization through the HIF-1α/VEGF signaling pathway. The specific mechanism is that it inhibits the secretion of VEGF in ARPE-19 cells under hypoxic conditions by downregulating the protein expression of HIF-1α and PHD-2 [60]. This process helps inhibit abnormal new blood vessel growth, which could play a role in treating DR. In summary, we speculated that the components in CQLT work together in various ways to exert their anti-DR effects.

GO enrichment analysis yielded 1597 BPs, primarily encompassing responses to hormones, positive regulation of cell migration, inflammatory responses, and responses to nutrient levels. Meanwhile, because quercetin, kaempferol, and formononetin in CQLT are mainly anti-DR through anti-inflammatory effects, we speculated that the inflammatory response is the most important biological process of CQLT for DR treatment. In addition, we used animal experiments to investigate the effects of CQLT on inflammatory cytokine to verify our speculation further. AGE-RAGE signaling pathway in diabetic complications, HIF-1 signaling pathway, and calcium signaling pathway were strongly associated with DR by KEGG pathway enrichment analysis. Based on the PPI network and the component-target-pathway network, we selected IL-6, TNF-α, VEGF, HIF-1α, and mTOR for study. Molecular docking results demonstrated that CQLT presented good binding affinity for these targets. Numerous experimental studies have revealed that the HIF-1 signaling pathway is crucial in DR [61, 62]. IL-6, VEGF, HIF-1 α, and mTOR are the core target proteins in this signaling pathway. Continuous oxygen supply is necessary for normal tissue development, dynamic balance, and function in all eukaryotes. The response to hypoxia is mediated by HIF-1, a heterodimeric complex composed of an oxygen-dependent subunit (HIF-1α) and a constitutively expressed nuclear subunit (HIF-1β) [63]. HIF-1α is expressed in all tissues and can contribute significantly to the generation of neovascularization by directly increasing the transcription of VEGF. Blocking or silencing the HIF-1α/VEGF signaling pathway has been reported to have potential benefits for DR [64]. Meanwhile, inhibition of HIF-1α overexpression in the retina of DR rats normalized the levels of IL-6 and TNF-α [65]. The VEGF protein family includes VEGF (or VEGF-A), VEGF-B, VEGF-C, *etc*. [66]. VEGF can promote endothelial cell migration and proliferation in retinal microvessels, increase vascular permeability, and participate in the process of DR neovascularization [67]. Because patients with DR are often associated with hypoxia, inflammation, oxidative stress, and other pathological processes, they stimulate overexpression of VEGF, and the level of VEGF is related to the severity of DR [68]. Interleukin-6 (IL-6) is a cytokine with multiple biological functions and is closely linked to inflammation in the body [69]. Individuals with DM tend to have elevated IL-6 levels in their bloodstream, which consequently promotes the development of DR [70]. IL-6 not only directly promotes angiogenesis but also induces the expression of VEGF to promote neovascularization, thus promoting the development of DR [71]. A meta-analysis study examined the IL-6 levels between DR patients and healthy controls and found that DR patients had significantly higher IL-6 levels than controls [72]. TNF-α, an essential mediator in the inflammatory response, is predominantly produced by monocytes and macrophages. It participates in the occurrence and development of DR by increasing the permeability of retinal blood vessels, stimulating excessive production of the extravascular matrix, and proliferation of vascular cells, leading to intraocular neovascularization [73]. Mammalian Target of Rapamycin (mTOR) is a conserved serine/threonine protein kinase that plays a critical role in the pathogenesis and progression of DR [74]. Blocking STZ-induced retinal overexpression of mTOR in DR rats inhibited the HIF-1α/VEGF signaling pathway, thereby alleviating cell damage during DR development and beneficially impacting DR pathogenesis [75].

The thinning of retinal thickness may accelerate the progression of DR, and several studies have found that the thinning of retinal thickness in DR patients and experimental animals is due to retinal cell death [76, 77]. In the present study, retinal thickness was significantly increased in the CQLT group of rats compared with the model group. Moreover, histological examination of retinal tissues revealed that CQLT could markedly alleviate major characteristics of DR, including retinal exudation, neovascularization, and vacuolar degeneration of cells. These results demonstrated that CQLT alleviated the severity of DR lesions and improved retinal pathological damage, thereby exerting protective effects on the retina. After CQLT intervention, the DR rats showed significantly decreased expression of retinal mTOR, HIF-1α, VEGF, IL-6, and TNF-α, along with markedly reduced serum levels of IL-6 and TNF-α. Taken together, our study preliminarily elucidated that CQLT exhibited certain preventive and therapeutic effects on DR in ZDF rats. The mechanisms of action involved CQLT inhibiting activation of the mTOR/HIF-1α/VEGF signaling pathway, reducing expression of the inflammatory cytokine IL-6 and TNF-α, suppressing neovascularization, and increasing retinal thickness, thereby exerting anti-DR effects.

The CQLT active ingredients screened based on the web database were screened according to the oral bioavailability and drug-like properties methods, which may have missed some low content but powerful ingredients, resulting in an incomplete result, and we plan to analyze further the CQLT ingredients using mass spectrometry and high-performance liquid chromatography (HPLC) in the future. In addition, based on the preliminary research work, we plan to conduct clinical trials to evaluate the efficacy and safety of CQLT in human DR patients, study the synergistic effects of the individual components in CQLT, and elucidate its exact mechanism of action.

## CONCLUSION

In this study, the mechanisms of action of CQLT in treating DR were systematically investigated through network pharmacology, molecular docking, and *in vivo* experiments. The results showed that quercetin, kaempferol, and formononetin were the important active components in CQLT. CQLT exerts against DR effects by inhibiting the mTOR/HIF-1α/VEGF signaling pathway, alleviating inflammatory response, and suppressing neovascularization in the retina, thereby protecting retinal function and morphology. These results reflected the positive impact of CQLT on the occurrence and development of DR through multi-component, multi-target, and multi-pathway. The results of our study provide the theoretical foundation and experimental basis for the clinical application of CQLT in the treatment of DR, as well as research ideas for the subsequent development of herbs for the treatment of DR.

## Figures and Tables

**Fig. (1):**
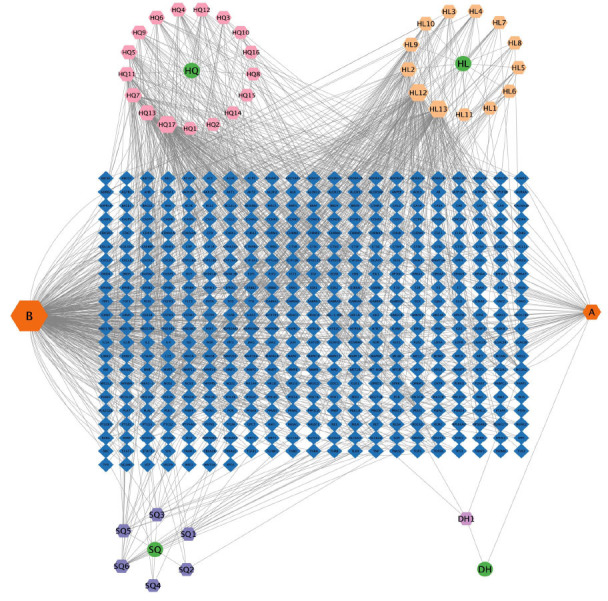
Herb-component-target network. The blue diamond nodes represent the targets of CQLT, the green circle nodes represent the four herbs of CQLT, and the hexagonal nodes of different colors represent the components. The pink, light yellow, purple, and light purple nodes represent the components of Astragalus membranaceus (HQ), Coptis chinensis (HL), Panax notoginseng (SQ), and Rehmannia glutinosa (DH), respectively. The orange nodes A represents stigmasterol, a common component of DH and SQ, and B represents quercetin, a common component of HQ, HL, and SQ.

**Fig. (3):**
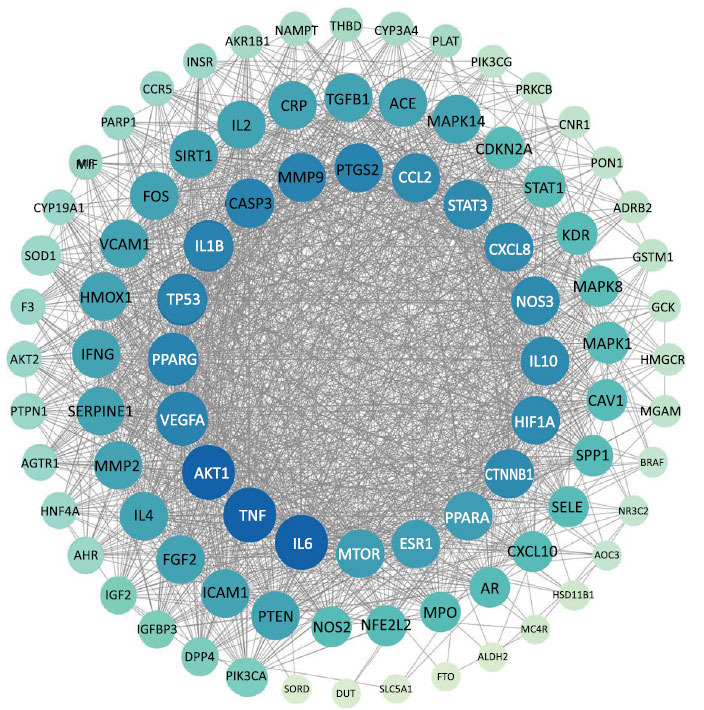
PPI network of CQLT targets against DR. Nodes with larger sizes and darker colors had higher degree values.

**Fig. (4):**
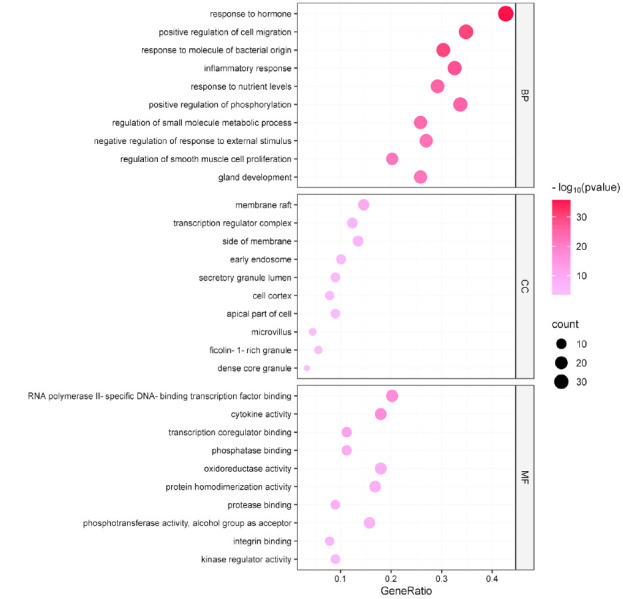
The top 10 BP, CC, and MF terms of GO enrichment analysis.

**Fig. (7):**
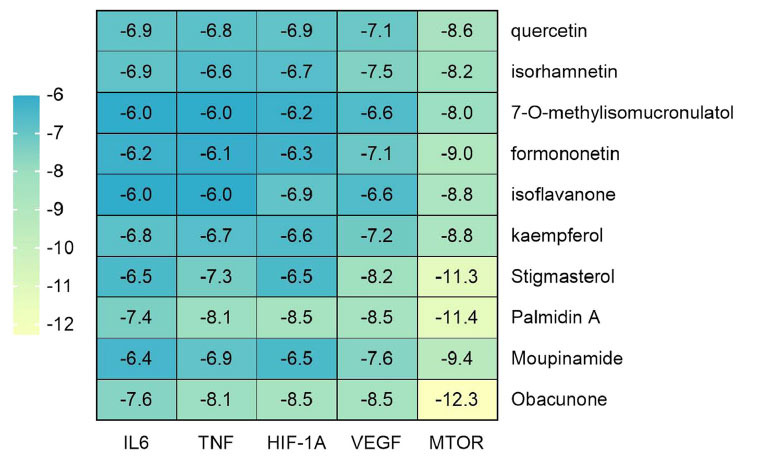
Binding energy heatmap of docking between active components and core targets.

**Fig. (9):**
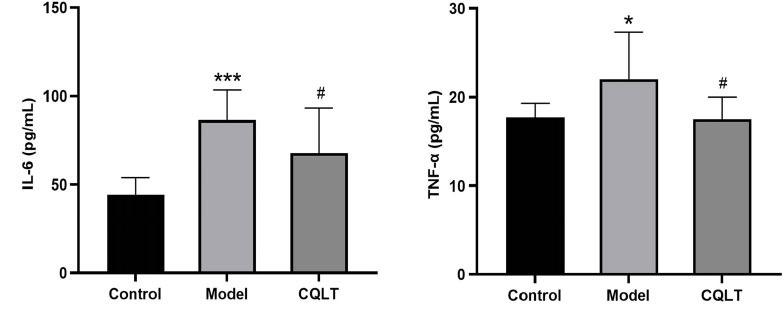
The levels of serum IL-6 and TNF- α were evaluated by ELISA. *P < 0.05, ***P < 0.01 *vs*. the control group; #*P* < 0.05 *vs*. the model group.

**Fig. (11):**
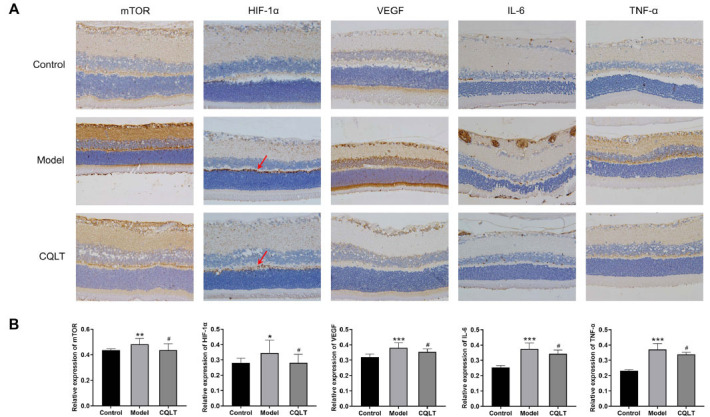
Immunohistochemical analysis of the retina. **(A)** Effects of CQLT on mTOR, HIF-1α, VEGF, IL-6, and TNF-α in the retina of DR rats. The magnification was 400×,. **(B)** The results of immunohistochemistry were statistically analyzed using the NIS-ELEMNT BR image analysis system. *P < 0.05, **P < 0.01, ***P < 0.01 *vs*. the control group; #*P* < 0.05 *vs*. the model group.

**Table 1:** Information table of key components of CQLT.

**MOL ID**	**Component Name**	**Degree Value**
MOL000098	quercetin	432
MOL000398	isoflavanone	101
MOL013352	Obacunone	101
MOL008647	Moupinamide	101
MOL000422	kaempferol	57
MOL000449	Stigmasterol	56
MOL000378	7-O-methylisomucronulatol	40
MOL000762	Palmidin A	35
MOL000392	formononetin	34
MOL000354	isorhamnetin	29

## Data Availability

The datasets used and/or analyzed during the current study are available from the corresponding authors upon reasonable request.

## LIST OF ABBREVIATIONS

BP: Biological Process
CC: Cellular Component
CQLT: Compound Qilian Tablets
DM: Diabetes Mellitus
DR: Diabetic Retinopathy
GO: Gene Ontology
HE: hematoxylin and Eosin
IL-6: Interleukin-6
KEGG: Kyoto Encyclopedia of Genes and Genomes
MF: Molecular Function
mTOR: Mammalian Target of Rapamycin
PBS: Phosphate Buffered Saline
PPI: Protein-protein Interaction
SPF: Specific Pathogen-free
TCM: Traditional Chinese Medicine
TNF-α: Tumor Necrosis Factor-alpha
VEGF: Vascular Endothelial Growth Factor
ZDF: Zucker Diabetic Fatty
