# Supplementary material for: Network Pharmacology and Molecular Docking to Explore the Mechanism of Compound Qilian Tablets in Treating Diabetic Retinopathy
Source: Curr Comput Aided Drug Des. 2024 Mar 15;21(3):333–47. doi: 10.2174/0115734099298932240308104437 (PMC12272071; doi:10.2174/0115734099298932240308104437)
Supplement: Supplementary file 1 [file CCADD-21-3-333_SD1.zip › CCADD-21-3-333_SD1/CCADD-SUPPLY-2023-335.pdf]

SUPPLEMENTARY MATERIAL

Network Pharmacology and Molecular Docking to Explore the Mechanism of Compound Qilian Tablets in Treating Diabetic Retinopathy

Jiangwei Jia[1,2], Bo Liu[1], Xin Wang[1], Fenglan Ji[1], Fuchun Wen[1], Lianlian Song[1], Huibo Xu[1,*] and Tao Ding[1,*]

[1]*Pharmacodynamic and Toxicological Evaluation Center, Jilin Academy of Chinese Medicine Sciences, Changchun, Jilin, China;* [2]*School of Pharmaceutical Sciences, Changchun University of Chinese Medicine, Changchun, Jilin, China*

Table S1. Information table of the herb-component-target network.

Herb	Mark	MOL ID	Component Name
Rehmannia glutinosa (Dihuang)	DH1	MOL000359	sitosterol
	A	MOL000449	Stigmasterol
Panax notoginseng (Sanqi)	B	MOL000098	quercetin
	A	MOL000449	Stigmasterol
	SQ1	MOL000358	beta-sitosterol
	SQ2	MOL001494	Mandenol
	SQ3	MOL001792	DFV
	SQ4	MOL002879	Diop
	SQ5	MOL005344	ginsenoside rh2
	SQ6	MOL007475	ginsenoside f2
Astragalus membranaceus (Huangqi)	B	MOL000098	quercetin
	HQ1	MOL000033	(3S,8S,9S,10R,13R,14S,17R)-10,13-dimethyl-17-[(2R,5S)-5-propan-2-yloctan-2-yl]-2,3,4,7,8,9,11,12,14,15,16,17-dodecahydro-1H-cyclopenta[a]phenanthren-3-ol
	HQ2	MOL000211	Mairin
	HQ3	MOL000239	Jaranol
	HQ4	MOL000296	hederagenin
	HQ5	MOL000354	isorhamnetin
	HQ6	MOL000371	3,9-di-O-methylnissolin
	HQ7	MOL000378	7-O-methylisomucronulatol
	HQ8	MOL000379	9,10-dimethoxypterocarpan-3-O-β-D-glucoside
	HQ9	MOL000380	(6aR,11aR)-9,10-dimethoxy-6a,11a-dihydro-6H-benzofurano[3,2-c]chromen-3-ol

	HQ10	MOL000387	Bifendate
	HQ11	MOL000392	formononetin
	HQ12	MOL000417	Calycosin
	HQ13	MOL000422	kaempferol
	HQ14	MOL000433	FA
	HQ15	MOL000439	isomucronulatol-7,2'-di-O-glucosiole
	HQ16	MOL000442	1,7-Dihydroxy-3,9-dimethoxy pterocarpene
	HQ17	MOL000398	isoflavanone
Coptis chinensis (Huanglian)	B	MOL000098	quercetin
	HL1	MOL000622	Magnograndiolide
	HL2	MOL000762	Palmidin A
	HL3	MOL000785	palmatine
	HL4	MOL001454	berberine
	HL5	MOL001458	coptisine
	HL6	MOL002668	Worenine
	HL7	MOL002894	berberrubine
	HL8	MOL002897	epiberberine
	HL9	MOL002903	(R)-Canadine
	HL10	MOL002904	Berlambine
	HL11	MOL002907	Corchoroside A_qt
	HL12	MOL008647	Moupinamide
	HL13	MOL013352	Obacunone

Table 2. Information table of the protein-protein interaction network.

Name	Degree	BetweennessCentrality	ClosenessCentrality
IL6	148	0.040408872	0.862745098
TNF	148	0.041713469	0.862745098
AKT1	148	0.036758226	0.862745098
VEGFA	142	0.025635756	0.838095238
PPARG	136	0.057813886	0.814814815
TP53	132	0.023820366	0.8
IL1B	130	0.018275331	0.792792793
CASP3	122	0.012708674	0.765217391

MMP9	118	0.009029322	0.739495798
PTGS2	118	0.010140732	0.745762712
CCL2	116	0.01043493	0.745762712
STAT3	116	0.012515173	0.727272727
CXCL8	114	0.008225357	0.721311475
NOS3	114	0.017090553	0.739495798
IL10	112	0.009797273	0.733333333
HIF1A	110	0.006524945	0.721311475
CTNNB1	108	0.007787127	0.709677419
PPARA	104	0.032041718	0.704
ESR1	102	0.008876246	0.698412698
MTOR	100	0.008407509	0.692913386
PTEN	100	0.008885689	0.682170543
ICAM1	100	0.00730293	0.698412698
FGF2	98	0.004854071	0.676923077
IL4	98	0.007415251	0.682170543
MMP2	98	0.004262749	0.676923077
HMOX1	96	0.005861268	0.682170543
SERPINE1	96	0.007102249	0.676923077
IFNG	96	0.003965656	0.676923077
SIRT1	94	0.008700915	0.682170543
FOS	94	0.008222065	0.666666667
VCAM1	94	0.006171284	0.676923077
CRP	92	0.009261477	0.666666667
IL2	92	0.006778668	0.671755725
ACE	90	0.011794659	0.671755725
TGFB1	90	0.001944187	0.656716418
MAPK14	88	0.003234362	0.651851852
CDKN2A	84	0.011429982	0.647058824
STAT1	82	0.001796712	0.637681159
KDR	80	0.002563551	0.633093525
MAPK1	80	0.013413284	0.633093525

CAV1	80	0.005526982	0.637681159
MAPK8	80	0.002260228	0.633093525
SPP1	78	0.003031623	0.628571429
SELE	70	0.001179027	0.606896552
AR	68	0.003998159	0.602739726
CXCL10	68	0.001127932	0.606896552
MPO	66	0.002203641	0.602739726
NFE2L2	66	0.002729594	0.611111111
NOS2	66	0.00112018	0.611111111
DPP4	64	0.02010684	0.611111111
PIK3CA	64	0.00250397	0.594594595
IGFBP3	60	9.85E-04	0.586666667
IGF2	60	0.003532465	0.590604027
AHR	56	0.001849364	0.586666667
AGTR1	52	0.001789781	0.575163399
HNF4A	52	0.00208564	0.578947368
PTPN1	52	0.003002118	0.586666667
F3	50	2.97E-04	0.560509554
SOD1	50	0.022424096	0.582781457
AKT2	50	7.92E-04	0.571428571
CYP19A1	46	0.002174369	0.564102564
CCR5	44	6.65E-04	0.556962025
INSR	44	0.003450513	0.560509554
MIF	44	3.11E-04	0.556962025
PARP1	44	2.71E-04	0.556962025
AKR1B1	42	0.018571982	0.567741935
THBD	40	1.26E-04	0.536585366
NAMPT	40	1.80E-04	0.553459119
CYP3A4	38	0.002821197	0.556962025
PLAT	36	2.61E-05	0.530120482
PIK3CG	32	4.26E-04	0.530120482
PRKCB	30	0.001215238	0.536585366

CNR1	28	3.54E-04	0.533333333
PON1	26	5.32E-04	0.530120482
ADRB2	26	4.61E-04	0.526946108
GSTM1	24	0.004091273	0.517647059
HMGCR	22	1.29E-04	0.523809524
GCK	22	0.004631972	0.520710059
MGAM	20	0.003085647	0.514619883
NR3C2	20	0.001281981	0.511627907
BRAF	20	1.62E-04	0.511627907
AOC3	18	0.003055103	0.5
HSD11B1	14	2.89E-04	0.50867052
MC4R	12	7.21E-04	0.486187845
ALDH2	8	4.94E-04	0.403669725
FTO	6	6.02E-05	0.47311828
SLC5A1	6	0	0.391111111
SORD	4	0	0.380952381
DUT	4	0	0.451282051
